# *Methylenetetrahydrofolate reductase* tagging polymorphisms are associated with risk of non-small cell lung cancer in eastern Chinese Han population

**DOI:** 10.18632/oncotarget.22887

**Published:** 2017-12-04

**Authors:** Hao Ding, Yafeng Wang, Yuanmei Chen, Chao Liu, Hao Qiu, Mingqiang Kang, Weifeng Tang

**Affiliations:** ^1^ Department of Respiratory Disease, Affiliated People's Hospital of Jiangsu University, Zhenjiang, Jiangsu Province, China; ^2^ Department of Cardiology, The People's Hospital of Xishuangbanna Dai Autonomous Prefecture, Jinghong, Yunnan Province, China; ^3^ Department of Thoracic Surgery, Fujian Cancer Hospital, Fujian Medical University Cancer Hospital, Fuzhou, Fujian Province, China; ^4^ Department of Cardiothoracic Surgery, Affiliated People's Hospital of Jiangsu University, Zhenjiang, Jiangsu Province, China; ^5^ Department of Immunology, School of Medicine, Jiangsu University, Zhenjiang, Jiangsu Province, China; ^6^ Department of Thoracic Surgery, Fujian Medical University Union Hospital, Fuzhou, Fujian Province, China

**Keywords:** MTHFR, polymorphism, non-small cell lung cancer, risk

## Abstract

Previous reports implicated 5,10-*ethylenetetrahydrofolate reductase* (*MTHFR*) polymorphisms acted as a potential risk factor for several cancers. In order to explore the effect of *MTHFR* SNPs on non-small cell lung cancer (NSCLC), we selected *MTHFR* tagging single nucleotide polymorphisms (SNPs) and carried out a case-control study to determine the potential relationship of *MTHFR* SNPs with NSCLC risk. Our study consisted of 521 NSCLC patients and 1,030 non-cancer controls. *MTHFR* SNPs were genotyped by SNPscan^TM^ genotyping assay. Using four genetic models (additive, Homozygote, dominant, recessive), the genotype frequencies were compared using the chi-squared (*χ*^2^) test. Crude/adjusted odds ratios (ORs) with their 95% confidence intervals (CIs) were used to assess the difference for the genotype distribution. We found that MTHFR rs1801133 G>A polymorphism decreased the risk of overall NSCLC. In a subgroup analysis, *MTHFR* rs1801133 G>A polymorphism also decreased NSCLC risk in female, < 60 years and never smoking subgroups. However, we identified that MTHFR rs4845882 G>A polymorphism was associated with the development of NSCLC in female subgroup. In addition, *MTHFR* rs9651118 T>C polymorphism increased the risk of NSCLC in < 60 years, never smoking and BMI < 24 kg/m^2^ subgroups. In conclusion, the current study highlights *MTHFR* rs1801133 G>A variants decreases the risk of NSCLC. Nevertheless, MTHFR rs4845882 G>A and rs9651118 T > C polymorphisms may be associated with NSCLC susceptibility. Well-designed large-scale studies are needed to confirm these findings and explore the interactions of gene-gene and gene-environment involved in *MTHFR* SNPs and NSCLC.

## INTRODUCTION

Lung cancer (LC) caused by multiple risk factors is one of the common malignancies worldwide. With very complex biological characteristics and high degree of invasiveness, it is difficult to diagnose at an early stage and lack of very effective treatment at an advanced stage. Thus, LC is a common public health problem with a poor prognosis. LC involves two major subtypes, such as small cell LC and non-small cell LC (NSCLC). In addition, NSCLC cases account for most of the total LC cases. The increasing incidence of NSCLC is closely related to tobacco consumption, air pollution, cooking fumes, asbestos and other environmental factors [1]. However, these known risk factors might not contribute to overall susceptibility to NSCLC. Recently, individual's genetic factors have also been determined to cause NSCLC.

Folic acid, or 5-methyltetrahydrofolate, is a cofactor in the metabolism of homocysteine to methionine [2]. 5,10-ethylenetetrahydrofolate reductase (MTHFR) catalyzed reduction of 5,10-methylenetetrahydrofolate (methylene-THF), a donor of methyl for dUMP to dTMP transform, to methyl-THF, the primary methyl donor in methionine synthesis. Methionine is transformed to S-adenosyl-l-methionine (SAM) which is the principal methyl donor in over 100 biochemical responses, including cytosine methylation in DNA. By the catalysis of DNA (cytosine-5)-methyltransferase, methyl group of SAM was transferred to C5 of cytosine within CpG island in the genomic DNA in higher eukaryotes [3–5]. MTHFR is a dimeric flavoprotein in human and each monomer is bound to flavinadenosine-dinucleotide noncovalently [6]. Each monomer contains a N-terminal catalytic domain that binds the allosteric SAM inhibitory regulating enzyme activity in response to the methionine levels in the cell [7].The findings of the relationship between methylation patterns and folate status in individuals with cancer and healthy normal individuals provide stronger evidences for a mechanism by which folate may modify DNA methylation and alter the risk of cancer.

Methylenetetrahydrofolate reductase (MTHFR), whose gene maps to the short arm of Chromosome 1 and encodes a 77-kDa protein with 656 amino acids. Many single nucleotide polymorphisms (SNPs) have been identified (http://www.ncbi.nlm.nih.gov/SNP), such as rs1801131, rs1801133, rs1537514, rs9651118, rs1537516, rs3753584, rs4845882, rs4846048, rs2066462 and rs3737967 polymorphisms, etc. A number of case-control studies focused on the association between *MTHFR* polymorphisms and the risk of LC [8–15], however, the results were inconsistent. For example, a meta-analysis suggested that *MTHFR* rs1801133 G>A was not associated the risk of LC in Chinese population [16]. Nevertheless, Yang *et al.* reported that *MTHFR* rs1801133 G>A polymorphism increased the risk of lung cancer in Asians, but not in Caucasians [17]. These ambiguous findings may be due to the limited sample size or difference in populations. In order to extensively explore the relationship of *MTHFR* SNPs with LC susceptibility, we selected *MTHFR* tagging SNPs (rs3753584 T>C, rs4845882 G>A, rs1801133 G>A, rs4846048 A>G and rs9651118 T>C) and carried out a case-control study to determine the potential effect of *MTHFR* SNPs on NSCLC risk.

## RESULTS

### Baseline characteristics

In this study, a total of 521 sporadic NSCLC patients and 1,030 normal controls were enrolled. Age and sex were full matched (*P* = 0.843 and *P* = 0.453, respectively; Table 1). Of the NSCLC patients, 287 were male and 234 were female, with a mean age of 59.76 ± 10.71 years. The non-cancer controls were comprised of 588 males and 442 females with a mean age of 60.34 ± 9.11 years. Of the tobacco consumption and drinking and body mass index (BMI), differences were found between NSCLC patients and non-cancer controls (*P* < 0.001, Table 1). The genotype distribution of *MTHFR* was calculated after genotyping the 1,551 included participants. For *MTHFR* rs1801133 G>A, rs4845882 G>A, rs4846048 A>G, rs3753584 T>C and rs9651118 T>C polymorphisms, success rates of genotyping were 99.87%, 99.94%, 99.94%, 99.94% and 99.94%, respectively (Table 2). The genotype distribution of *MTHFR* SNPs reached Hardy–Weinberg equilibrium (HWE) in controls, except for *MTHFR* rs4846048 A>G polymorphism (*P* = 0.036) (Table 2).

**Table 1:** Distribution of selected demographic variables and risk factors in NSCLC cases and controls

Variable	Overall Cases (*n* = 521)	Overall Controls (*n* = 1,030)	*P*^a^
	*n* (%)	*n* (%)	
Age (years)	59.76 ± 10.71	60.34 ± 9.11	0.268
Age (years)			0.843
< 60	238 (45.68)	476 (46.21)	
≥ 60	283 (54.32)	554 (53.79)	
Sex			0.453
Male	287 (55.09)	588 (57.09)	
Female	234 (44.91)	442 (42.91)	
Smoking status			< 0.001
Never	317 (60.84)	828 (80.39)	
Ever	204 (39.16)	202 (19.61)	
Alcohol use			**< 0.001**
Never	444 (85.22)	949 (92.14)	
Ever	77 (14.78)	81 (7.86)	
BMI (kg/m^2^)	23.00 (± 3.03)	23.84 (± 3.06)	**< 0.001**
BMI (kg/m^2^)			
< 24	337 (64.68)	547 (53.11)	**< 0.001**
≥ 24	184 (35.32)	483 (46.89)	

^a^ Two-sided *χ*^2^ test and Student *t* test

**Table 2:** Primary information for *MTHFR* polymorphisms (rs1801133 G>A, rs9651118 T>C rs4845882 G>A, rs4846048 A>G and rs3753584 T>C)

Genotyped SNPs	rs1801133 G>A	rs3753584 T>C	rs4845882 G>A	rs4846048 A>G	rs9651118 T>C
Chromosome	1	1	1	1	1
Function	Missense	NearGene-5	Intron	Intron	Intron
Chr Pos (Genome Build 36.3)	11778965	11787173	11765754	11768839	11784801
MAF^a^ for Chinese in database	0.439	0.093	0.198	0.105	0.382
MAF in our controls (***n =*** 1,030)	0.345	0.118	0.214	0.095	0.383
*P* value for HWE^b^ test in our controls	0.947	0.712	0.454	0.036	0.081
Genotyping method	SNPscan	SNPscan	SNPscan	SNPscan	SNPscan
% Genotyping value	99.87%	99.94%	99.94%	99.94%	99.94%

^a^MAF: minor allele frequency;

^b^HWE: Hardy–Weinberg equilibrium

### Association of *MTHFR* rs1801133 G>A, rs4845882 G>A, rs4846048 A>G, rs3753584 T>C and rs9651118 T>C polymorphisms with the development of NSCLC

Table 3 summarizes the genotypes of *MTHFR* SNPs. *MTHFR* rs1801133 G>A polymorphism decreased the risk of NSCLC in two genetic models [AA *vs.* GG: crude odds ratio (OR) = 0.66, 95% confidence interval (CI): 0.45–0.96, *P* = 0.031; and AA *vs.* GA/GG: crude OR = 0.69, 95% CI: 0.48–0.99, *P* = 0.042; Table 3]. Adjustment for age, sex, BMI, smoking and drinking, the decreased risk of NSCLC was also found (AA *vs.* GG: adjusted OR = 0.66, 95% CI: 0.47–0.97, *P* = 0.035; Table 3). However, the above findings were not significant after the Bonferroni correction for multiple comparisons. For *MTHFR* rs3753584 T>C, rs4845882 G>A, rs4846048 A>G and rs9651118 T>C polymorphisms, we found null association between these SNPs and the risk of NSCLC (Table 3).

**Table 3:** Logistic regression analyses of associations between *MTHFR* rs1801133 G>A, rs3753584 T>C, rs4845882 G>A, rs4846048 A>G and rs9651118 T>C polymorphisms and the risk of NSCLC

Genotype	Cases(*n* = 521)		Controls(*n* = 1,030)		Crude OR(95%CI)	*P*	Adjusted OR ^a^(95%CI)	*P*
	*n*	%	*n*	%				
***MTHFR* rs1801133 G>A**								
GG	241	46.35	441	42.86	1.00		1.00	
GA	235	45.19	466	45.29	0.92 (0.74–1.15)	0.467	0.92 (0.73–1.16)	0.461
AA	44	8.46	122	11.86	**0.66 (0.45–0.96)**	**0.031**	**0.66 (0.44–0.97)**	**0.035**
GA + AA	279	53.65	588	57.14	0.87 (0.70–1.07)	0.192	0.87 (0.70–1.08)	0.207
GG+ GA	476	91.54	907	88.14	1.00		1.00	
AA	44	8.46	122	11.86	**0.69 (0.48–0.99)**	**0.042**	0.69 (0.47–1.00)	0.050
A allele	323	31.06	710	34.50				
***MTHFR* rs3753584 T>C**								
TT	403	77.35	800	77.75	1.00		1.00	
CT	111	21.31	216	20.99	1.02 (0.79–1.32)	0.872	1.03 (0.79–1.35)	0.829
CC	7	1.34	13	1.26	1.07 (0.42–2.71)	0.885	1.04 (0.39–2.76)	0.937
CT+CC	118	22.65	229	22.25	1.02 (0.80–1.32)	0.860	1.03 (0.79–1.34)	0.826
TT+CT	514	98.66	1,016	98.74	1.00		1.00	
CC	7	1.34	13	1.26	1.07 (0.42–2.69)	0.894	1.03 (0.39–2.74)	0.948
C allele	125	12.00	242	11.76				
***MTHFR* rs4845882 G>A**								
GG	309	59.31	632	61.42	1.00		1.00	
GA	191	36.66	354	34.40	1.11 (0.89–1.38)	0.378	1.12 (0.89–1.42)	0.326
AA	21	4.03	43	4.18	1.00 (0.58–1.72	0.999	1.14 (0.65–2.01)	0.642
GA+AA	212	40.69	397	38.58	1.09 (0.88–1.35)	0.422	1.12 (0.90–1.10)	0.308
GG+GA	500	95.97	986	95.82	1.00		1.00	
AA	21	4.03	43	4.18	0.96 (0.57–1.64)	0.891	1.09 (0.63–1.91)	0.753
A allele	233	22.36	440	21.38				
***MTHFR* rs4846048 A>G**								
AA	428	82.15	849	82.51	1.00		1.00	
AG	90	17.27	165	16.03	1.08 (0.82–1.44)	0.578	1.13 (0.84–1.51)	0.423
GG	3	0.58	15	1.46	0.40 (0.11–1.38)	0.146	0.48 (0.13–1.73)	0.264
AG+GG	93	17.85	180	17.49	1.03 (0.78–1.35)	0.861	1.08 (0.81–1.44)	0.609
AA+AG	518	99.42	1,014	98.54	1.00		1.00	
GG	3	0.58	15	1.46	0.39 (0.11–1.36)	0.140	0.47 (0.13–1.70)	0.250
G allele	96	9.21	195	9.48				
***MTHFR* rs9651118 T>C**								
TT	187	35.89	378	36.73	1.00		1.00	
TC	245	47.02	513	49.85	0.97 (0.77–1.22)	0.783	0.94 (0.74–1.20)	0.636
CC	89	17.08	138	13.41	1.31 (0.95–1.80)	0.100	1.30 (0.93–1.81)	0.124
TC+CC	334	64.11	651	63.27	1.04 (0.83–1.29)	0.745	1.02 (0.81–1.28)	0.895
TT+TC	432	82.92	891	86.59	1.00		1.00	
CC	89	17.08	138	13.41	1.33 (1.00–1.78)	0.054	1.34 (0.99–1.82)	0.057
C allele	423	40.60	789	38.34				

^a^ Adjusted for age, sex, smoking, BMI and drinking status; Bold values are statistically significant (*P* < 0.05).

### Association of *MTHFR* rs1801133 G>A, rs4845882 G>A, rs4846048 A>G, rs3753584 T>C and rs9651118 T>C polymorphisms with the development of NSCLC in Different Stratification Groups

After adjustment by logistic regression analysis, we found *MTHFR* rs1801133 G>A variants were associated with the decreased risk of NSCLC in some subgroups (female group: AA *vs.* GG: adjusted OR = 0.53, 95% CI 0.30–0.94, *P* = 0.031 and AA *vs.* GA/GG: adjusted OR = 0.58, 95% CI 0.33–1.00, *P* = 0.048; < 60 years subgroup: AA *vs.* GG: adjusted OR = 0.53, 95% CI 0.28–1.00, *P* = 0.048; never smoking group: AA *vs.* GG: adjusted OR = 0.58, 95% CI 0.36–0.93, *P* = 0.024 and AA *vs.* GA/GG: adjusted OR = 0.62, 95% CI 0.39–0.99, *P* = 0.044; Table 4).

**Table 4:** Stratified analyses between *MTHFR* rs1801133 G>A polymorphism and NSCLC risk by sex, age, BMI, smoking status and alcohol consumption

Variable	MTHFR rs1801133 G>A (case/control)^a^			Adjusted OR^b^ (95% CI); *P*				
	GG	GA	AA	GG	GA	AA	GA /AA	AA vs. (GA/GG)
Sex								
Male	125/254	136/275	25/58	1.00	0.99 (0.72–1.35);*P*: 0.930	0.83 (0.48–1.45);*P*: 0.518	0.97 (0.71–1.32);*P*: 0.840	0.84 (0.50–1.43);*P*: 0.527
Female	116/187	99/191	19/64	1.00	0.85 (0.61–1.20);*P*: 0.357	**0.53 (0.30–0.94);P: 0.031**	0.78 (0.56–1.07);*P*: 0.126	**0.58 (0.33–1.00);P: 0.048**
Age								
< 60	125/213	97/213	16/49	1.00	0.79 (0.56–1.12);*P*: 0.187	**0.53 (0.28–1.00);P: 0.048**	0.74 (0.53–1.03);*P*: 0.072	0.59 (0.32–1.09);*P*: 0.090
≥ 60	116/228	138/253	28/73	1.00	1.05 (0.77–1.44);*P*: 0.759	0.78 (0.47–1.30);*P*: 0.344	1.01 (0.74–1.36);*P*: 0.976	0.77 (0.47–1.24);*P*: 0.276
Smoking status								
Never	153/352	137/372	26/103	1.00	0.86 (0.65–1.14);*P*: 0.298	**0.58 (0.36–0.93);P: 0.024**	0.81 (0.62–1.05);*P*: 0.112	**0.62 (0.39–0.99);P: 0.044**
Ever	88/89	98/94	18/19	1.00	1.06 (0.70–1.60);*P*: 0.786	0.92 (0.45–1.90);*P*: 0.829	1.04 (0.70–1.54);*P*: 0.861	0.90 (0.45–1.78);*P*: 0.754
Alcohol consumption								
Never	210/411	197/426	36/111	1.00	0.93 (0.72–1.19);*P*: 0.543	0.68 (0.44–1.03);*P*: 0.071	0.88 (0.70–1.12);*P*: 0.293	0.71 (0.47–1.06);*P*: 0.092
Ever	31/30	38/40	8/11	1.00	0.88 (0.44–1.75);*P*: 0.711	0.60 (0.21–1.75);*P*: 0.353	0.82 (0.42–1.58);*P*: 0.550	0.65 (0.24–1.75);*P*: 0.393
BMI (kg/m^2^)								
< 24	159/242	151/247	27/57	1.00	0.94 (0.70–1.27);*P*: 0.699	0.68 (0.41–1.15);*P*: 0.149	0.89 (0.67–1.19);*P*: 0.427	0.70 (0.43–1.16);*P*: 0.164
≥ 24	82/199	84/219	17/65	1.00	0.88 (0.61–1.27);*P*: 0.490	0.64 (0.35–1.17);*P*: 0.147	0.84 (0.59–1.19);*P*: 0.323	0.69 (0.38–1.23);*P*: 0.203

^a^ The genotyping was successful in 521 (99.81%) NSCLC cases, and 1030 (99.90%) controls for *MTHFR* rs1801133 G>A;

^b^ Adjusted for age, sex, BMI, smoking status and alcohol consumption (besides stratified factors accordingly) in a logistic regression model;

The correlation between *MTHFR* rs3753584 T>C polymorphism and NSCLC risk in the stratified analyses are summarized Table 5. We found that *MTHFR* rs3753584 T>C vatiants were not associated with the susceptibility of NSCLC in any subgroup (Table 5).

**Table 5:** Stratified analyses between *MTHFR* rs3753584 T>C polymorphism and NSCLC risk by sex, age, BMI, smoking status and alcohol consumption

Variable	MTHFR rs3753584 T>C (case/control)^a^			Adjusted OR^b^ (95% CI); *P*				
	TT	TC	CC	TT	TC	CC	TC / CC	CC vs. (TC/TT)
Sex								
Male	231/450	52/127	4/10	1.00	0.80 (0.54–1.17);*P*: 0.242	0.68 (0.20–2.32);*P*: 0.533	0.78 (0.54–1.14);*P*: 0.201	0.71 (0.21–2.42);*P*: 0.578
Female	172/350	59/89	3/3	1.00	1.35 (0.92–1.97);*P*: 0.128	2.51 (0.479–12.75);*P*: 0.268	1.38 (0.95–2.01);*P*: 0.093	2.34 (0.46–11.88);*P*: 0.304
Age								
< 60	181/362	54/110	3/3	1.00	0.95 (0.65–1.40);*P*: 0.806	1.30 (0.25–6.67);*P*: 0.756	0.96 (0.66–1.41);*P*: 0.848	1.31 (0.26–6.73);*P*: 0.745
≥ 60	222/438	57/106	4/10	1.00	1.12 (0.77–1.63);*P*: 0.560	0.89 (0.26–3.04);*P*: 0.855	1.10 (0.76–1.58);*P*: 0.610	0.87 (0.26–2.97);*P*: 0.827
Smoking status								
Never	240/650	73/168	4/9	1.00	1.16 (0.84–1.60);*P*: 0.359	1.48 (0.44–5.00);*P*: 0.528	1.18 (0.86–1.61);*P*: 0.314	1.43 (0.43–4.83);*P*: 0.562
Ever	163/150	38/48	3/4	1.00	0.77 (0.47–1.25);*P*: 0.281	0.66 (0.14–3.02);*P*: 0.593	0.76 (0.47–1.21);*P*: 0.247	0.70 (0.15–3.20);*P*: 0.645
Alcohol consumption								
Never	341/735	96/202	7/11	1.00	0.99 (0.74–1.32);*P*: 0.937	1.43 (0.52–3.92);*P*: 0.489	1.01 (0.76–1.33);*P*: 0.951	1.43 (0.52–3.92);*P*: 0.486
Ever	62/65	15/14	0/2	1.00	1.31 (0.56–3.07);*P*: 0.537	-	1.08 (0.48–2.45);*P*: 0.852	-
BMI (kg/m^2^)								
< 24	265/432	69/105	3/9	1.00	1.04 (0.73–1.48);*P*: 0.830	0.47 (0.12–1.84);*P*: 0.278	0.99 (0.70–1.40);*P*: 0.960	0.47 (0.12–1.82);*P*: 0.272
≥ 24	138/368	42/111	4/4	1.00	0.99 (0.65–1.50);*P*: 0.946	3.34 (0.80–14.04);*P*: 0.100	1.06 (0.70–1.59);*P*: 0.788	3.35 (0.80–14.04);*P*: 0.098

^a^ The genotyping was successful in 521 (100.00%) NSCLC cases, and 1030 (99.90%) controls for *MTHFR* rs3753584 T>C;

^b^ Adjusted for age, sex, BMI, smoking status and alcohol consumption (besides stratified factors accordingly) in a logistic regression model;

The relationship of *MTHFR* rs4845882 G>A polymorphism with NSCLC susceptibility in the stratified analysis is listed in Table 6. We identified that *MTHFR* rs4845882 G>A polymorphism was associated with the development of NSCLC in female subgroup (GA *vs.* GG: adjusted OR = 1.47, 95% CI 1.05–2.05, *P* = 0.025).

**Table 6:** Stratified analyses between *MTHFR* rs4845882 G>A polymorphism and NSCLC risk by sex, age, BMI, smoking status and alcohol consumption

Variable	MTHFR rs4845882 G>A (case/control)^a^			Adjusted OR^b^ (95% CI); *P*				
	GG	GA	AA	GG	GA	AA	GA /AA	AA vs. (GA/GG)
Sex								
Male	177/349	94/212	16/26	1.00	0.87 (0.63–1.21);*P*: 0.411	1.42 (0.71–2.85);*P*: 0.322	0.93 (0.68–1.26);*P*: 0.627	1.49 (0.75–2.96);*P*: 0.254
Female	132/283	97/142	5/17	1.00	1.47 (1.05–2.05);P: 0.025	0.68 (0.24–1.90);*P*: 0.460	1.39 (1.00–1.93);*P*: 0.051	0.59 (0.21–1.63);*P*: 0.304
Age								
<60	136/286	92/169	10/20	1.00	1.17 (0.83–1.65);*P*: 0.361	1.15 (0.50–2.61);*P*: 0.746	1.17 (0.84–1.63);*P*: 0.359	1.08 (0.48–2.42);*P*: 0.862
≥60	173/346	99/185	11/23	1.00	1.07 (0.78–1.47);*P*: 0.685	1.12 (0.52–2.43);*P*: 0.777	1.07 (0.79–1.46);*P*: 0.653	1.09 (0.51–2.35);*P*: 0.820
Smoking status								
Never	186/510	119/282	12/35	1.00	1.16 (0.88–1.53);*P*: 0.301	1.09 (0.54–2.18);*P*: 0.815	1.15 (0.88–1.51);*P*: 0.313	1.03 (0.52–2.04);*P*: 0.940
Ever	123/122	72/72	9/8	1.00	1.02 (0.68–1.55);*P*: 0.914	1.21 (0.45–3.27);*P*: 0.705	1.04 (0.70–1.56);*P*: 0.842	1.20 (0.45–3.20);*P*: 0.714
Alcohol consumption								
Never	260/581	168/327	16/40	1.00	1.14 (0.89–1.45);*P*: 0.312	0.99 (0.53–1.85);*P*: 0.982	1.12 (0.88–1.42);*P*: 0.354	0.95 (0.51–1.75);*P*: 0.858
Ever	49/51	23/27	5/3	1.00	0.95 (0.47–1.92);*P*: 0.893	2.23 (0.47–10.67);*P*: 0.315	1.06 (0.54–2.08);*P*: 0.858	2.27 (0.48–10.64);*P*: 0.298
BMI (kg/m^2^)								
< 24	207/338	120/190	10/18	1.00	1.06 (0.78–1.42);*P*: 0.724	1.03 (0.45–2.35);*P*: 0.952	1.05 (0.79–1.41);*P*: 0.737	1.00 (0.44–2.28);*P*: 0.992
≥ 24	102/294	71/164	11/25	1.00	1.24 (0.85–1.79);*P*: 0.262	1.26 (0.58–2.72);*P*: 0.557	1.24 (0.87–1.77);*P*: 0.236	1.16 (0.55–2.48);*P*: 0.697

^a^ The genotyping was successful in 521 (100.00%) NSCLC cases, and 1030 (99.90%) controls for *MTHFR* rs4845882 G>A;

^b^ Adjusted for age, sex, BMI, smoking status and alcohol consumption (besides stratified factors accordingly) in a logistic regression model;

Table 7 demonstrated that *MTHFR* rs4846048 A>G polymorphism was not associated with the development of NSCLC in any subgroup.

**Table 7:** Stratified analyses between *MTHFR* rs4846048 A>G polymorphism and NSCLC risk by sex, age, BMI, smoking status and alcohol consumption

Variable	*MTHFR* rs4846048 A>G (case/control)^a^			Adjusted OR^b^ (95% CI); *P*				
	AA	AG	GG	AA	AG	GG	AG/GG	GG vs. (AG/AA)
Sex								
Male	233/488	51/88	3/11	1.00	1.29(0.86-1.94);*P*: 0.221	0.68(0.18-2.64);*P*: 0.576	1.22(0.83-1.81);*P*: 0.314	0.65(0.17-2.52);*P*: 0.535
Female	195/361	39/77	0/4	1.00	0.97(0.63-1.49);*P*: 0.884	-	0.92(0.60-1.41);*P*: 0.701	-
Age								
< 60	191/393	47/74	0/8	1.00	1.45(0.95-2.22);*P*: 0.088	-	1.32(0.87-2.00);*P*: 0.197	-
≥ 60	237/456	43/91	3/7	1.00	0.88(0.59-1.33);*P*: 0.555	0.94(0.23-3.86);*P*: 0.936	0.89(0.60-1.32);*P*: 0.558	0.96(0.24-3.93);*P*: 0.959
Smoking status								
Never	262/676	53/140	2/11	1.00	0.98(0.69-1.40);*P*: 0.925	0.60(0.13-2.79);*P*: 0.512	0.96(0.68-1.36);*P*: 0.810	0.60(0.13-2.79);*P*: 0.513
Ever	166/173	37/25	1/4	1.00	1.54(0.89-2.68);*P*: 0.126	0.31(0.03-2.87);*P*: 0.302	1.39(0.81-2.36);*P*: 0.230	0.29(0.03-2.67);*P*: 0.274
Alcohol consumption								
Never	366/779	76/156	2/13	1.00	1.06(0.77-1.45);*P*: 0.723	0.41(0.09-1.89);*P*: 0.254	1.01(0.75-1.38);*P*: 0.931	0.41(0.09-1.87);*P*: 0.248
Ever	62/70	14/9	1/2	1.00	1.95(0.77-4.96);*P*: 0.162	0.57(0.05-7.23);*P*: 0.664	1.70(0.71-4.09);*P*: 0.238	0.52(0.04-6.52);*P*: 0.610
BMI(kg/m^2^)								
< 24	281/455	55/86	1/5	1.00	1.10(0.75-1.62);*P*: 0.629	0.55(0.06-4.92);*P*: 0.591	1.08(0.74-1.57);*P*: 0.708	0.54(0.06-4.83);*P*: 0.579
≥ 24	147/394	35/79	2/10	1.00	1.20(0.76-1.89);*P*: 0.433	0.41(0.08-2.02);*P*: 0.274	1.10(0.71-1.71);*P*: 0.678	0.40(0.08-1.96);*P*: 0.258

^a^The genotyping was successful in 521 (100.00%) NSCLC cases, and 1030 (99.90%) controls for *MTHFR* rs4846048 A>G;

^b^Adjusted for age, sex, BMI, smoking status and alcohol consumption (besides stratified factors accordingly) in a logistic regression model.

We found that *MTHFR* rs9651118 T>C polymorphism increased the risk of NSCLC in several stratified analyses (<60 years group: CC *vs.* TT: adjusted OR = 1.64, 95% CI 1.00–2.69, *P* = 0.049 and CC *vs.* TC/TT: adjusted OR = 1.75, 95% CI 1.12–2.74, *P* = 0.014; never smoking subgroup: CC *vs.* TC/TT: adjusted OR = 1.50, 95% CI 1.05–2.14, *P* = 0.025; BMI < 24 kg/m^2^ group: CC *vs.*TT: adjusted OR = 1.56, 95% CI 1.01–2.39, *P* = 0.044 and CC *vs.* TC/TT: adjusted OR = 1.56, 95% CI 1.06–2.29, *P* = 0.023; Table 8).

**Table 8:** Stratified analyses between *MTHFR* rs9651118 T>C polymorphism and NSCLC risk by sex, age, BMI, smoking status and alcohol consumption

Variable	*MTHFR* rs9651118 T>C (case/control)^a^			Adjusted OR^b^ (95% CI); *P*				
	TT	TC	CC	TT	TC	CC	TC / CC	CC vs. (TC/TT)
Sex								
Male	110/209	133/300	44/78	1.00	0.85 (0.61–1.18);*P*: 0.330	1.11 (0.69–1.76);*P*: 0.676	0.90 (0.66–1.23);*P*: 0.503	1.21 (0.79–1.86);*P*: 0.381
Female	77/169	112/213	45/60	1.00	1.05 (0.73–1.51);*P*: 0.794	1.48 (0.92–2.39);*P*: 0.109	1.15 (0.81–1.61);*P*: 0.435	1.44 (0.94–2.22);*P*: 0.098
Age								
<60	82/163	110/255	46/57	1.00	0.90 (0.62–1.29);*P*: 0.549	1.64 (1.00–2.69);P: 0.049	1.03 (0.73–1.45);*P*: 0.868	1.75 (1.12–2.74);P: 0.014
≥60	105/215	135/258	43/81	1.00	1.00 (0.72–1.39);*P*: 1.00	1.05 (0.66–1.65);*P*: 0.848	1.01 (0.74–1.38);*P*: 0.946	1.05 (0.69–1.59);*P*: 0.834
Smoking status								
Never	111/305	146/412	60/110	1.00	0.93 (0.69–1.25);*P*: 0.646	1.45 (0.98–2.14);*P*: 0.066	1.04 (0.79–1.37);*P*: 0.791	1.50 (1.05–2.14);P: 0.025
Ever	76/73	99/101	29/28	1.00	0.95 (0.62–1.46);*P*: 0.819	0.97 (0.52–1.80);*P*: 0.918	0.96 (0.64–1.44);*P*: 0.824	1.00 (0.56–1.76);*P*: 0.989
Never	159/340	205/479	80/129	1.00	0.87 (0.67–1.13);*P*: 0.296	1.25 (0.88–1.78);*P*: 0.212	0.95 (0.74–1.21);*P*: 0.677	1.35 (0.99–1.86);*P*: 0.062
Ever	28/38	40/34	9/9	1.00	1.66 (0.83–3.30);*P*: 0.153	1.45 (0.49–4.24);*P*: 0.501	1.61 (0.83–3.11);*P*: 0.155	1.11 (0.40–3.03);*P*: 0.845
BMI (kg/m^2^)								
< 24	108/187	165/287	64/72	1.00	0.99 (0.72–1.36);*P*: 0.967	1.56 (1.01–2.39);P: 0.044	1.10 (0.81–1.49);*P*: 0.532	1.56 (1.06–2.29);P: 0.023
≥ 24	79/191	80/226	25/66	1.00	0.85 (0.58–1.25);*P*: 0.406	0.95 (0.55–1.64);*P*: 0.850	0.87 (0.61–1.25);*P*: 0.456	1.03 (0.62–1.72);*P*: 0.908

^a^The genotyping was successful in 521 (100.00%) NSCLC cases, and 1030 (99.90%) controls for *MTHFR* rs9651118 T>C;

^b^Adjusted for age, sex, BMI, smoking status and alcohol consumption (besides stratified factors accordingly) in a logistic regression model;

### SNP haplotypes

Using SHEsis software (http://analysis.bio-x.cn) [18], we further constructed seven *MTHFR* haplotypes (Table 9). Haplotype comparison analysis indicated that *MTHFR* haplotypes were not associated with the risk of NSCLC.

**Table 9:** *MTHFR* haplotype frequencies (%) in cases and controls and risk of NSCLC

	Case (*n* = 1042)		Control (*n* = 2060)		Crude OR (95% CI)	*P*
	*n*	%	*n*	%		
G T G A C	414	39.81	774	37.63	1.00	
A T G A T	317	30.48	687	33.40	0.86 (0.72–1.03)	0.105
G C A A T	121	11.63	220	10.70	1.03 (0.80–1.32)	0.828
G T A G T	92	8.85	185	8.99	0.93 (0.70–1.23)	0.606
G T G A T	68	6.54	131	6.37	0.97 (0.71–1.33)	0.853
G T A A T	13	1.25	21	1.02	1.16 (0.57–2.34)	0.683
Others	15	1.44	39	1.90	0.72 (0.39–1.32)	0.285

With the order of rs1801133 G>A, rs3753584 T>C, rs4845882 G>A, rs4846048 A>G and rs9651118 T>C in gene position.

## DISCUSSION

In the present study, the relationships of *MTHFR* tagging polymorphisms with the development of NSCLC risk were explored. The results highlighted that *MTHFR* rs1801133 G>A might decrease the risk of overall NSCLC. In addition, we found *MTHFR* rs1801133 G>A variants were associated with the decreased risk of NSCLC in female, < 60 years and never smoking subgroups. However, we found that *MTHFR* rs4845882 G>A polymorphism was associated with the development of NSCLC in female subgroup. The association between *MTHFR* rs9651118 T>C polymorphism and the increased the risk of NSCLC was also evident in < 60 years, never smoking and BMI < 24 kg/m^2^ subgroups.

Variants of *MTHFR*, which is an important regulator of intracellular folate metabolism, were found that they were associated with the increased level of circulating homocysteine and many diseases involving NSCLC. A number of case-control studies focused on the association of NSCLC with *MTHFR* SNPs and had controversial findings. Recently, a meta-analysis which included twenty-six studies demonstrated that *MTHFR* contribute to the risk of NSCLC in Asians and overall populations, but not Caucasians [17]. Another meta-analysis which enrolled 10 studies with 2487 cases and 3228 controls suggested that rs1801133 G>A polymorphism in *MTHFR* gene may not be a risk factor of NSCLC in Chinese populations; however, the association between this SNP and NSCLC risk might alter in different region of China [16]. Clearly, these ambiguous findings indicated that the function of *MTHFR* rs1801133 G>A polymorphism might be varied in different race or even in the different region of the same ethnicity, which suggested large-scale case-control studies in different regions and ethnicities were needed to further explore the potential relationship. It was found that activation and variant frequencies of *MTHFR* might alter among different region and different latitude with the various ultraviolet-exposure levels [19, 20]. Furthermore, the sample sizes were relatively small in most of included studies. A functional study indicated that *MTHFR* rs1801133 G>A polymorphism was a protective factor of prostate cancer (PC) susceptibility by elevating homocysteine level, promoting cell apoptosis, and inhibiting proliferation of PC cells [21]. In the present study, we found that *MTHFR* rs1801133 A allele might be a protective factor for NSCLC, which was similar to the findings of previous study conducted in Eastern Chinese Han populations. However, these potential associations were not significant after the Bonferroni correction for multiple comparisons. Thus, our findings should be explained with very cautions.

Rs4845882 G>A, a SNP locate in intron region of *MTHFR* gene, was strongly complete linkage disequilibrium (LD) with *MTHFR* rs1801131 A>C polymorphism [(*r*2 = 0.935); http://gvs.gs.washington.edu/GVS147/]. Wang *et al.* reported there was no significant correlation between *MTHFR* rs4845882 G>A polymorphism and gastric cardia carcinoma (GCA) risk [22]. However, another study saw a decreased esophageal squamous cell carcinoma (ESCC) risk in Chinese Han individuals with *MTHFR* rs4845882 AA genotype [23]. In the present study, we found that *MTHFR* 4845882 G>A might be a risk factor for NSCLC in female subgroup. These inconsistent findings may be due to the limited sample size or other confounding factors. In the future, large-scale study with comprehensive functional exploring should be conducted. And the confounding gene or environmental factors also could not be ignored.

In this study, we found *MTHFR* rs9651118 T>C polymorphism was associated with the NSCLC risk in some subgroups. And recent case-control studies indicated that this SNP might play different roles among different type of cancer. For example, some studies suggested that *MTHFR* rs9651118 T>C polymorphism was associated with the decreased susceptibility of LC and PC [14, 24]. While Tang *et al*. and Wang *et al.* observed a null association of *MTHFR* rs9651118 T>C polymorphism with the risk of ESCC and GCA [22, 23]. Therefore, whether the T-to-C transition in the intron 2 region does alter the functions of *MTHFR* gene needs to be further explored.

However, several limitations in our study must be acknowledged. Firstly, other functional SNP loci in the region of the *MTHFR* gene may be related to NSCLC susceptibility. Unfortunately, because of genotyping cost, we were unable to perform a fine-mapping study focusing on the association between *MTHFR* SNPs and NSCLC risk. Secondly, the sample size of NSCLC patients was moderate and detailed information of some NSCLC patients was not available. The relationship of *MTHFR* SNPs with tumor stages or cancer subtypes was not carried out. This could limit the validity of the findings because these potentially factors might not be well understood. Thirdly, selected biases might result in spurious findings because the NSCLC patients and the controls were enrolled from the local hospitals. Finally, other potential gene-environment factors were not considered. Further studies focusing on the interactions of multiple environment and gene factors on NSCLC risk are needed to confirm our findings.

In conclusion, the current study highlights *MTHFR* rs1801133 G>A variants are associated with the decreased risk of NSCLC. However, *MTHFR* rs4845882 G>A and *MTHFR* rs9651118 T>C polymorphisms may increase the risk of NSCLC. Well-designed large-scale studies are needed to confirm these findings and explore the interactions of gene-gene and gene-environment involved in *MTHFR* SNPs and NSCLC.

## MATERIALS AND METHODS

### Ethics Statement

This case-control study was conducted in Fujian and Jiangsu Province in Eastern of China. The ethical board approval from Ethics Committee of Fujian Medical University (Fuzhou, China) and Jiangsu University (Zhenjiang, China) was obtained, and all of the participants signed written informed consent.

### Subjects

All sporadic NSCLC patients were enrolled from the Affiliated Union Hospital of Fujian Medical University and the Affiliated People's Hospital of Jiangsu University. Our study consisted of 521 NSCLC patients (mean age 59.76 ± 10.71 years) from January 2014 to December 2016. The diagnosis was confirmed based on pathological findings. For comparison, 1,030 non-cancer controls (mean age 60.34 ± 9.11 years) were recruited from normal volunteers who conducted health check in the Physical Examination Center of these hospitals. The controls had no history of autoimmune disorders or personal malignancy, and were frequency well-matched to patients by age and sex. The included risk factors (tobacco consumption and drinking) and demographic details of the NSCLC patients and controls were obtained by using a structured questionnaire. The data are listed in Table 1.

### Preparation of genomic DNA

Lymphocytes were separated from EDTA-anticoagulated whole blood. Genomic DNA was carefully extracted using the Promega DNA kit (Promega, Madison, USA).

### SNP selection

SNPs were selected using Haploview 4.2 software and the HapMap database. Five haplotype-tagging SNPs of *MTHFR* gene (rs3753584 T>C, rs4845882 G>A, rs1801133 G>A, rs4846048 A>G and rs9651118 T>C) were selected, with MAF > 5%, call rate ≥ 95 %, HWE *P* ≥ 0.05 and pair-wise r^2^ < 0.8 for each SNP pair. In total, the five tagging SNPs were selected by spaning the entire *MTHFR* gene region (upstream and downstream extending 5 Kb, respectively). The primary information of the selected SNPs is presented in Table 2.

### Genotyping

All SNPs were genotyped using the SNPscan^TM^ genotyping assay (Genesky Biotechologies Inc., Shanghai, China), which is a double ligation and multiplex fluorescence PCR [25]. The accuracy of genotyping results were verified by reanalyzing the genotypes in 4% random samples.

### Statistical analysis

Age of NSCLC patients and controls was described as the mean ± deviation (SD). And a Student's *t*-test was used to examine the difference for age. The deviation from HWE was assessed using an online goodness-of-fit chi-squared test (http://ihg.gsf.de/cgi-bin/hw/hwa1.pl) in controls [26–32]. Using different genetic models (additive, homozygote, dominant, recessive), the genotype frequencies of the subjects were compared using the chi-squared (*χ*^2^) test. Multivariate logistic regression analysis was harnessed to assess the risk of mutant genotype with respect to wild type and considered established confounders such as age, sex, smoking, BMI and drinking status. Crude/adjusted ORs with their 95% CIs were used to assess the difference for the genotype distribution. All data were analyzed using SAS software (Version 9.4; SAS Institute Inc., Cary, NC, USA). SHESIS program (Bio-X Inc., Shanghai, China, http://analysis.bio-x.cn/myAnalysis.php)] [18] was used to construct haplotypes of *MTHFR* gene. The association of *MTHFR* haplotypes with NSCLC risk was estimated as crude ORs with the corresponding 95% CIs. In this study, the threshold for significance was *P* < 0.05 (two tailed). We used Bonferroni correction to perform multiple comparisons [33].
